# Periodontal maintenance care compliance at federal qualified health clinics: a pilot study on dental staff’ experiences while serving underserved communities

**DOI:** 10.21142/2523-2754-1301-2025-230

**Published:** 2025-03-03

**Authors:** Yessenia Valverde Ingersoll

**Affiliations:** 1 La Maestra Community Health Center, 217 Highland Ave, National City, San Diego, EE. UU. yvalverdeg@gmail.com San Diego EE. UU yvalverdeg@gmail.com

**Keywords:** periodontal disease, federal qualified health centers, periodontal maintenance, scaling, root planning, underserved communities, cuidado periodontal, clínicas federales, enfermedad periodontal, comunidades necesitadas, raspado radicular, alisado radicular

## Abstract

**Objective:**

The aim of this study is to determine the patient compliance at Federal Qualified Health Centers and the dental staff actions and experiences in oral health promotion within underserved communities.

**Material and Methods:**

An online survey was distributed through email via the National Network of Oral Health Access, to professional dental staff, including dentists, dental hygienists, dental assistant, front desk receptionists. Questions covered about management skills on scaling and root planning, knowledge of methods used in periodontal maintenance care recalls, and comprehension of factors that can affect periodontal care compliance. Statistical analysis was done using proportion and frequency.

**Results:**

Subjects responded that 66% of patients are irregular compliant to periodontal care. Dental staff use education in periodontal health (25-38%), and behavior management (50%) tools to instruct patients during scaling and root planning. Conversely, receptionists use education in periodontal disease (50%) among prompt check-in, and financial assistance. All dental staff in FQHC, including receptionists, explain benefits of periodontal care and acknowledge concerns to continue patients' maintenance care recalls. For non-compliant patients, all dental staff used actions as visual education and explanation of consequences of lack of care, and receptionists (66%) responded that transferring phone calls was the most effective tool as patients responded better when contacted by their providers.

**Conclusion:**

In Federal Qualified Health Centers, active communication with patients and knowledge and education in periodontal disease, by dental staff and front desk receptionists have a favorable influence in patients periodontal care compliance.

## RESUMEN

**Objetivo:**

El objetivo de este estudio es determinar el cumplimiento de los pacientes en los Centros de Salud Federales Calificados y las experiencias y acciones del personal dental en la promoción de la salud bucal dentro de comunidades desatendidas.

**Material y métodos:**

Se distribuyó una encuesta en línea por correo electrónico, a través de la Red Nacional de Acceso a la Salud Bucal, al personal dental profesional, incluidos dentistas, higienistas dentales, asistentes dentales y recepcionistas. Se cubrieron preguntas sobre habilidades de gestión en raspado y alisado radicular, conocimiento de los métodos utilizados en los retiros del cuidado de mantenimiento periodontal y comprensión de los factores que pueden afectar el cumplimiento del cuidado periodontal. El análisis estadístico se realizó mediante proporción y frecuencia.

**Resultados:**

Los sujetos respondieron que el 66% de los pacientes tienen un cumplimiento irregular del cuidado periodontal. El personal dental utiliza herramientas de educación en salud periodontal (25-38%) y manejo del comportamiento (50%) para instruir a los pacientes durante el raspado y alisado radicular. Por el contrario, los recepcionistas utilizan la educación sobre la enfermedad periodontal (50%) entre el registro rápido y la asistencia financiera. Todo el personal dental de FQHC, incluidas las recepcionistas, explican los beneficios del cuidado periodontal y atienden las inquietudes de los pacientes. Para los pacientes que no cumplían, el personal dental utilizó acciones como educación visual y explicación de las consecuencias de la falta de atención, mientras que los recepcionistas (66%) respondieron que transferir llamadas telefónicas era la herramienta más efectiva, pues los pacientes respondían mejor cuando eran contactados por proveedores.

**Conclusión:**

En los Centros de Salud Calificados Federales, la comunicación activa con los pacientes y el conocimiento y educación en enfermedad periodontal, por parte del personal odontológico y recepcionistas, tiene una influencia favorable en el cumplimiento del cuidado periodontal de los pacientes.

## INTRODUCTION

Over the last 60 years, health centers have grown to become the cornerstone of community-based primary health care in the United States^1^. Over these years, they have become a beacon of hope, and a crucial portal to health care services, where medical underserved, high -risk patients, uninsured, refugees, asylees, and low-income communities seek medical and dental attention. These patients often exhibit chronic dental conditions, social issues, economic problems, and health disparities, and most of the time lack oral health knowledge for the prevention of systemic conditions^2^. 

Previous research discussed that the spread of oral bacteria in the mouth can contribute to inflammatory situations. In the 2000’s, the U.S. surgeon general affirmed for the first time, that oral health is important to general health. ^(^^3^^,^^4^ After this, several researchers found possible associations between oral disease and major systemic conditions. ^(^^5^ For instance, periodontal disease was identified as the sixth complication of diabetes by LÖe et al as early as 1993. ^(^^6^

Considering these important findings, systematic reviews found that globally most patients with major health conditions have poor knowledge and awareness of the oral health associations to their systemic condition. These systematic reviews particularly identified the lack of knowledge in oral health care in patients with heart disease, bone disease, and diabetes^7^^,^^5^. The majority of included studies indicated that ineffective health practitioner communication with their patients, regarding the oral-systemic link, is a predominant cause to both problems. Literacy on oral health care and oral health diseases is essential for these patients for the prevention of medical problems. Maintaining good oral care for prevention of periodontal diseases, prevents and helps to control diseases such as diabetes and hypertension. Therefore, it is crucial to maintain good periodontal care maintenance to reduce morbidity and to control systemic health conditions^8^. 

In the past, limited studies have reported on the factors that affect periodontal care maintenance among patients and dental staff. Some observations have been carried out in private offices, and it has been found that it takes participation from all staff in dental care to help patients in becoming more compliant to oral care^8^. Nevertheless, no studies have analyzed what actions are taken on the factors that affect periodontal maintenance care compliance in Federal Qualified Health Centers (FQHC.) 

Thus, the aim of this study was to assess periodontal care patient compliance in community health clinics and Federal Qualified Health Centers’ dental staff actions and experiences in periodontal oral health promotion within underserved communities.

## MATERIALS AND METHODS

For this study a qualitative survey research was chosen to collect the data. This research method helped to gather information of personal experiences by the dental staff explained in their own words and provided deeper insights from the staff when dealing with periodontal care maintenance patients. The ethical considerations included asking permission to send the survey to the list of members from National Network of Oral Health Access NNOHA, and explained that participation was voluntarily. No data, such as names or locations could be allocated with the survey link. 

This study participants included all professional dental caregivers, including dentists, dental hygienist, dental assistant, front desk, and receptionist's assistants within the National Network of Oral Health Access that work in federal qualified health centers. 

### 2.1. Data Collection

An online survey was distributed through email to all the participants by using SurveyMonkeyplatform (SurveyMonkey Inc.). To all participants that received the email with the survey link, it was specified that the participation in this survey was voluntarily and no data of their locations and names could be related to their answers. 

Questions were formulated based on the author’s own personal observation in clinics and after literature review from short communications articles written by hygienists^2^^,^^8^. No preliminary survey was found and a total of nine questions were formulated. Participants would take an average of five minutes to answer. The following questions included were:

1. What is your position?
2. What do you do to make patients feel at ease during SRP appointments? (Especially for those first timers)
3. What can you do to help guarantee that the patient will come back for their periodontal maintenance appointment after the SRP is completed?
4. What tools do you find useful to help convey the importance of oral care? (Select all that apply)
5. Which of the following could impact patient compliance to periodontal maintenance? (Select all that apply)
6. Do patients lack the ability to perceive the severity of periodontal disease? Please select and explain.
7. What are some reinforcement methods that you have found useful with non-compliant patients who don’t improve oral hygiene and don’t follow up with their appointments?
8. Overall, how do you rate your patient’s periodontal care compliance?
9. How can you motivate your patient(s) to do a better job of their oral hygiene?

Answers were reviewed and an open line-by-line coding was performed, where substantive codes reflecting the meaning of the data were identified and labeled concretely. Emerging codes with similar meanings were clustered into a common response code. 

The collection of data was done for a period of a month and collected by the author. The author strived to avoid being consciously governed by her own pre-structured understanding and to maintain a self-reflective attitude to ways in which the research process could be influenced and how this, in turn, could influence the research and answer analysis. 

*Data Management and Statistical Analysis*

All data were processed using the IBM SPSS Statistic 26 statistical package (IBM, SPSS Inc.). Analysis of answers was done by using coding, proportion and frequency. 

## RESULTS

### Participants

During the month of data collection, a total of 44 participants voluntarily participated and consented to answer to the survey. Due to small number of participants these preliminary data serve for this pilot study. ^(^^21^ Dental staff were classified as Thirteen dentists (including dentist and dental directors) 29%. Thirteen dental hygienists, 29%; Thirteen dental assistants, 29%; six front desk and clinic managers 14%. 

Dentist along with Dental Hygienist actively engage with patient during initial scaling root planning appointments in FQHC.

All dentists responded that they would educate patients about periodontal disease, 33%; and would engage patients with treatment expectations, 33%. The behavior management techniques used were: tell-show-do, 13% and rapport, 13%. All dentists discussed that listening was a great part of behavior management where patients’ concerns were addressed, 20%. 

**Figure 1:**
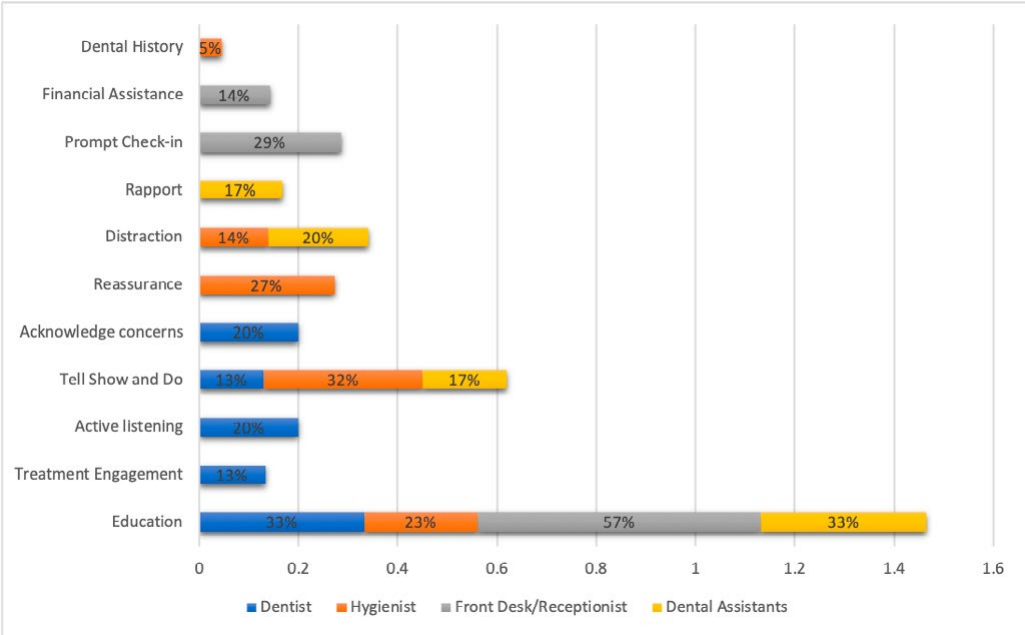
Strategies used by all dental staff in FQHC with patients during initial Scaling Root Planning appointment.

The Dental Hygienist, for instance, would be more actively engaged with patient and responded that they would use tell show and do techniques mainly when treating their patients, 32%; along with reassurance 27% and distraction techniques 14%. *“Encourage pts to bring headphones, wear comfortable clothing, get plenty of rest the night before, eat before coming. If I can, I ask beforehand what music they listen to, so I have that playing already”*. Hygienists also would educate on periodontal disease to the patients, 23%. *“Walk them through the entire procedure and answer all questions they may have”*. In addition to this, dental hygienists would also rely on dental and medical history review, 5%, to explain the importance of scaling and root planning to their patients (Figure 1).

Front Desk Receptionist staff in FQHC educate on Periodontal Disease during the initial Scaling Root Planning appointments

Front desk receptionist and dental managers responded that they would educate patients about periodontal disease 50% when making the appointments, and would provide prompted check-ins, 25% as well as financial assistance, 14%. Dental assistants lastly responded that they would explain treatment expectations to the patients undergoing scaling and root planning treatment (33%). They also explained that they would use behavior management techniques such as rapport (17%), distraction (33%) and tell show and do (17%), respectively (Figure 1).

Dental Hygienists in FQHC’s play an important role to help patients return for periodontal maintenance after scaling root planning is completed.

**Figure 2:**
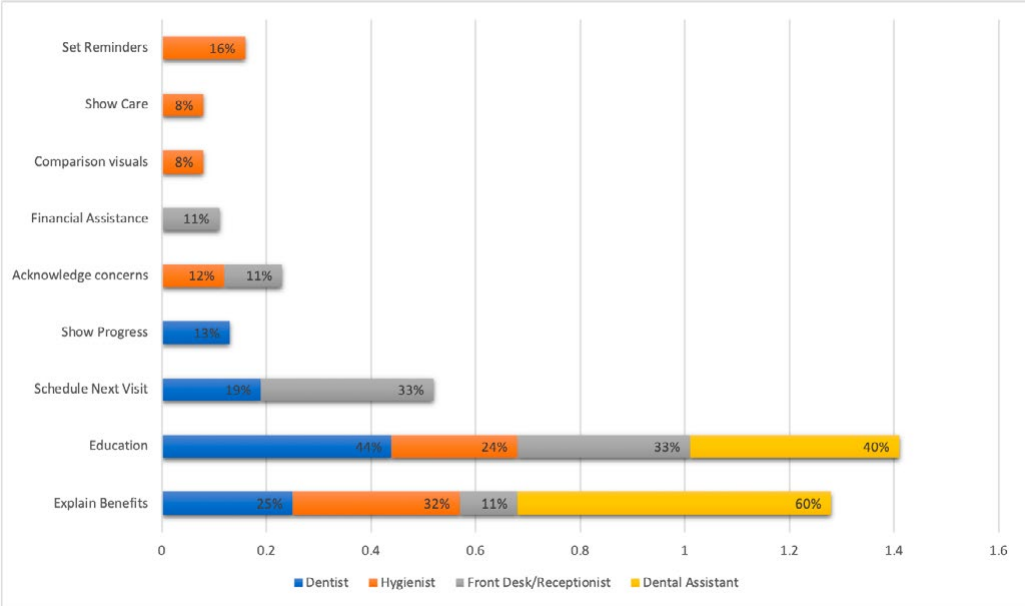
Dental Staff 's actions that help patients in FQHC return to Periodontal maintenance after Scaling and Root Planning is completed.

Dental hygienists responded that explanations of the benefits (32%) would be a useful tool to guarantee their patients would return to their periodontal maintenance appointments. Moreover, education on periodontal disease (24%) Setting reminders of the appointments (16%) and acknowledging concerns (12%) were also helpful. Comparison visuals (8%) from before and after is a reliable tool used by dental hygienist to motivate patients to come back for periodontal maintenance. Dentist also responded that they used comparison visual as showing the progress (13%) as important tool to help their patients return to periodontal maintenance care appointments in FQHC (See Figure 2). Dental assistants at the time of checking out patients, would use explanation of benefits (60%) as well as education of the periodontal disease (40%) to encourage patients return to their maintenance appointments (Figure 2). Front desk staff would use education on periodontal disease as well as prompt scheduling of the next appointments as useful actions to guarantee the patients periodontal care compliance. Additionally, the staff used as a reinforcement the explanation of the benefits, as well as acknowledging concerns over the phone, and would provide financial assistance in case insurance didn’t cover maintenance costs (Figure 2)

Intraoral pictures and x-rays demonstrating changes from before and after are useful to help convey the importance of oral care to patients being seen in Federal Health Centers.

**Figure 3:**
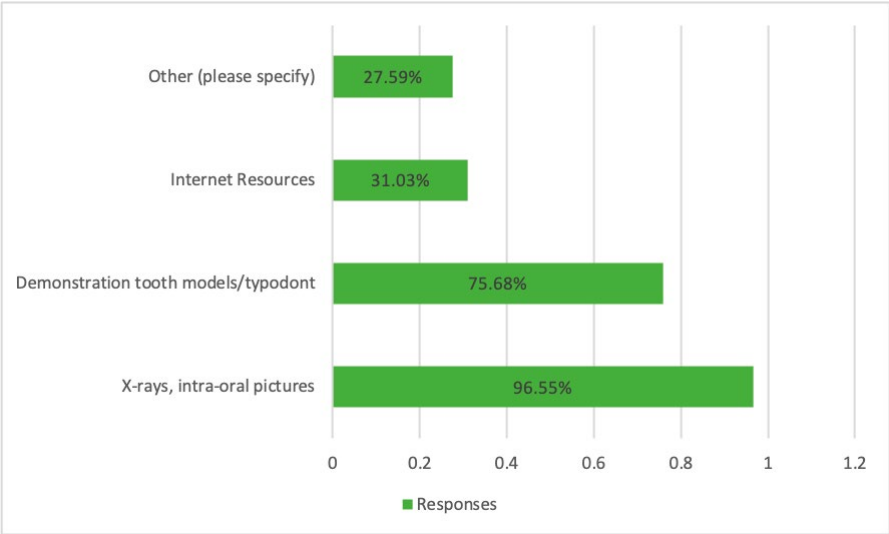
Tools that help convey oral care to patients in FQHC

A total of 96.55% of the responses indicated that dentist, dental hygienists, and dental assistants found the most useful tools to convey importance of periodontal care were to show intraoral photos as well as radiographs from before and after scaling and root planning. Demonstration by using typodonts tooth/models corresponded to 75.86% as the second most favorite tool used by the dental staff to convey periodontal care. The least used were Internet resources 31.03% and 27.59% corresponded to others, respectively (Figure 3)

Dental Staff in FQHC considered that no insurance coverage impacts greatly dental care compliance.

All the participants responded that not having financial resources as well as insurance coverage, 86.21%, respectively were seen as the main factors for discontinuation of periodontal care maintenance. In addition to these, the participants considered that substance abuse as well as education disparities 76.86% could affect compliance. 

Language barriers, 72.41% was also seen as a fifth factor that can interfere with periodontal maintenance. Housing problems were identified as the sixth factor along with mental illness and disabilities. Immigration status as well as lack of familiarity with the health care system were considered factors but could affect periodontal maintenance (Table 1)

**Table 1:** Factors that could impact periodontal care compliance in patients seen in FQHC.

Answer Choices	Responces		
Lack of financial resources	86.21 %	25
Mental illness	68.97 %	20
Disabilities	68.97 %	20
Education disparities	75.86 %	22
Language barriers	72.41 %	21
Lack of familiarity with healthcare system	58.62 %	17
Substance abuse	75.86 %	22
Housing problems	68.97 %	20
Immigration status	51.72 %	15
Insurance coverage limitations	86.21 %	25
Other (please specify)	17.24 %	5

3.6. Dental Staff in FQHC perceives patients lacking the ability to perceive the severity of periodontal disease.

**Figure 4:**
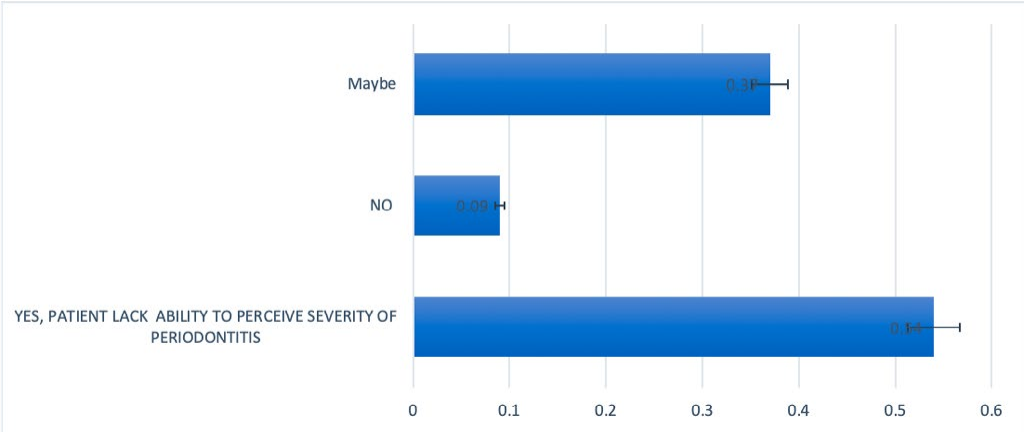
Perception from dental staff about patients’ ability to perceive severity of periodontal disease.

A total of 11 participants skipped this question but obtained answers from 35 participants. 54% of the participants responded to this question by stating that patients lacked the ability to perceive the severity of periodontal disease. Thirty seven percent (37%) replied that there is a possibility patients may not see the severity of periodontitis *"It really depends on the patient. Some patients completely comprehend the severity of their disease”*. Only 9% responded that they don’t believe patients lack the ability to perceive the severity of periodontal disease. (Figure 4)

3.7. Front Desk receptionist staff actions assist greatly with non- compliant patients in Federal Health Centers.

**Figure 5:**
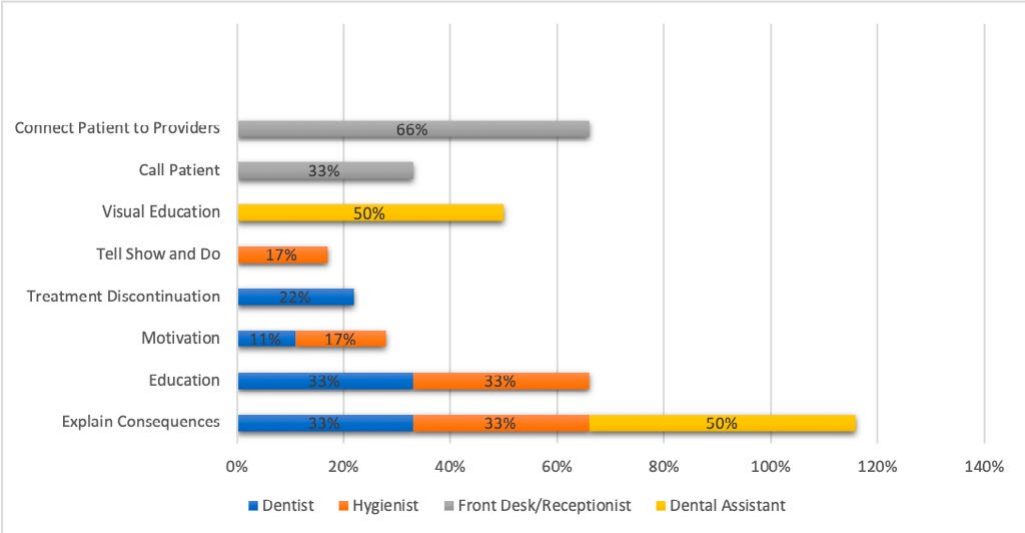
Reinforcement’s methods used by dental staff if patients discontinued periodontal care compliance in FQHC.

Among the reinforcement methods used by dentists with non -compliant patients, there is education (33%) and explanation of the consequences (33%) if patients weren’t compliant with periodontal care. Conversely, some practitioners would have to discontinue (22%) any additional restorative procedures if patients didn’t comply with periodontal maintenance care. Lastly, some would use motivation as a useful reinforcement method to improve compliance. (Figure 5) 

Dental hygienists on the other hand, as described in figure 5, responded that they would use education (33%) and explanation of the consequences (33%) if patients discontinued periodontal care. In small percentage, hygienist responded that they also use the tell, show and do (17%) technique as well as motivation (17%). In addition to this, dental assistants responded that they would use explanation of the consequences (50%) if patients lacked compliance, as well as visual education (50%) including internet photos.

Front desk receptionist and assistants also participated in this response and explained that they would call patients (33%) to remind them of periodontal maintenance recalls appointments. They also responded that they would have to connect patients with either the provider dentist (33%) or dental hygienist (33%) as patients responded better to show up to maintenance recalls when contacted by their own provider. *“I feel they respond better when allowing the hygienist or doctor to call. Patients respond better when the practitioner that works on them calls”*

Rate and classification of periodontal care maintenance patients in FQHC

**Figure 6:**
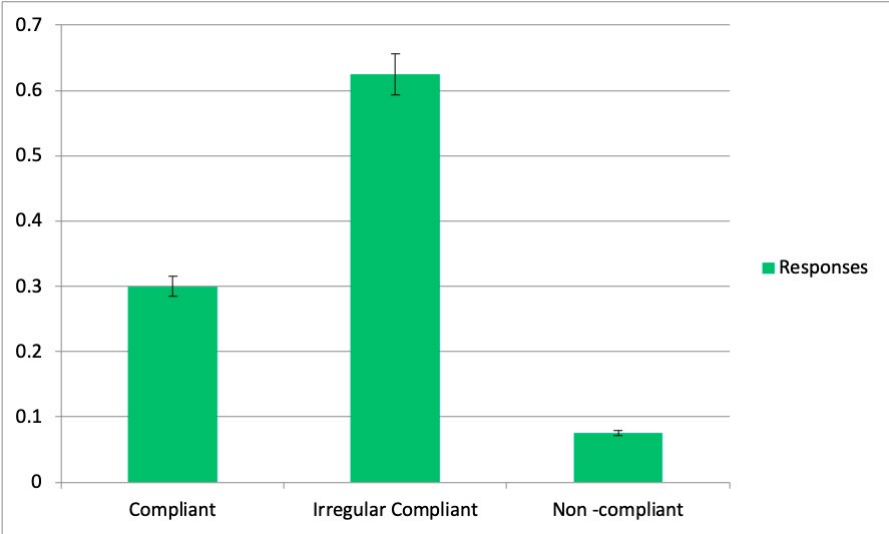
Patients’ classification in FQHC respective their compliance to periodontal care maintenance.

All dental staff survey participants responded that periodontal care maintenance patients are mostly irregular compliant (Figure 6). 

3.9. Dental Hygienists and Dental Assistants in FQHC thrive to promote periodontal care compliance in Federal Health Centers.

**Figure 7:**
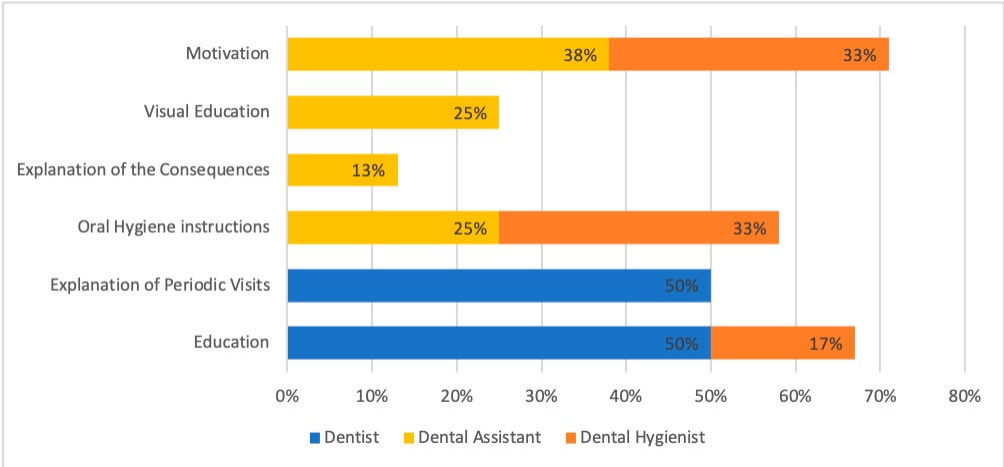
Techniques used to promote periodontal care compliance in FQHC patients.

All dentist, as described in Figure 7, would motivate their patients by using education on periodontal disease (50%) and by explaining the periodic visit benefits (50%).

The dental hygienist on the other hand would use more techniques, including motivation with visual aids (33%), oral hygiene instructions (33%), education on periodontal disease (17%), comparison visuals from pre-post op (17%), and re-enforcing the obtained improvements (17%) (Figure 7).

Dental assistants responded that they use explanation of the benefits of good oral care (38%), provide oral hygiene instructions (25%) and use visual education (25%). In a small percentage (13%) they use explanations of the consequences of lack of care. Figure 7.

## DISCUSSION

Periodontal maintenance and good oral health care begin with the patient’s treatment acceptance during the first appointment where scaling and root planning is done. ^7^^,^^8^^,^^9^ Great communication and ample discussion among patients and dental staff about periodontal disease etiology and periodontal disease therapy has been suggested^8^^,^^5^^).^ In our study all personnel in Federal Qualified Health Centers (FQHC), including receptionists would educate on periodontal disease at the time of the phone calls and check-ins, and would also engage the patients about treatment expectations to obtain the treatment acceptance in order for the patient to show up to their appointments. It has been recommended before that it takes all personnel participation from those that first greet the patients and to the doctor/hygienist for patient’s treatment acceptance. ^(^^8^^,^^2^^,^^11^

On the day of the procedure at FQHC; dental assistants, dentists, and dental hygienists would use mainly tell show and do technique in order to explain patients about periodontal disease and periodontal care therapy. In addition to this, only dental hygienists responded that other than the behavior management such as tell show and do, and social skills they would also rely on medical history review to enhance the importance of periodontal therapy. As described in an article by Jewell Goodman, the dental hygienists are well suited and would play a crucial role in community health centers when working next to other medical and dental providers. ^(^^2^ This is also good to bear in mind, because many patients aren’t aware of the connection between periodontal disease with other medical conditions. ^5^^,^^7^ It is relevant important to explain patients, especially when they are medically compromised, the correlation between periodontal health and systemic health.

A systematic review of randomized clinical trials and analytic studies of physician-patient communication confirmed a positive influence of quality communication on health outcomes. For a successful and humanistic encounter at an office visit, one needs to be sure that the patient's key concerns have been directly and specifically solicited and addressed. ^10^

Scaling and root planning can be very dreadful to some patients as it involves local anesthesia and multiple appointments for something that most patients considered a “teeth cleaning”. ^(^^2^^,^^8^ However, reinforcement of education on periodontal disease as well as showing the progress and comparison of visuals with radiographs and pictures from before and after treatment guaranteed that the patients in FQHC would return to maintenance appointments as described by our participants. In other studies, it has been elucidated the use of mail containing explanatory information in brochures with large pictures about periodontal disease to increase compliance and to increase the appointments show up rate. ^13^^,^^14^

Even though all personnel work to educate dental patients in FQHC, financial resources, as well as language barriers, may interfere with periodontal maintenance compliance. Our participants responded that financial resources were the number one reason that could cause patients to discontinue treatment. Studies have shown that to make dental treatments available and accessible, public dental health insurance should be expanded and should reach out more to low-income patients. ^(^^15^^,^^16^^,^^17^

Despite financial challenges, all dental staff personnel in FQHC work together to overcome this challenge. For instance, front desk staff would provide sliding fees whenever dental insurance wouldn’t cover anymore periodontal maintenance benefits. In addition to this, the rest of the staff would use reinforcement methods and explanation of the consequences if treatment is discontinued. The front desk receptionist actively sent phone calls reminders and sometimes patients would respond better when being contacted by their own provider. In a communication paper done by a hygienist, it is described that cell phone apps, and reminders could be great aids to motivate patients and remind of their periodontal maintenance appointments. ^8^

In our study not only staff receptionist sent reminders but also the dental hygienist and dental assistants appear to be involved in reminding and showing care to help patients to continue to show up to their appointments. Therefore, dental hygienists are a great staff to be part in FQHC to help promote oral health. 

Nevertheless, with all these efforts put together, patients seen for periodontal maintenance care in FQHC were categorized by dental staff as mostly irregular compliant. This could be due to varied reasons just as described before there could be other factors such as dental fear, anxiety that could cause discontinuation. In FQHC the load of patients are patients that can be categorized as difficult patients, since they present fear to the dentist ^12^^,^^15^. 

Survey respondents described that discontinuation could also be because patients may have had substance abuse disorders, education disparities and mental illness. These characteristics have also been described for difficult dental patients where is suggested to do more behavior management techniques for treatment acceptance and treatment continuation. ^(^^12^ The study however, reflected all participants’ experiences with periodontal maintenance care patients within FQHC network. This was only possible by including a survey method that contained open questions which enabled the dental staff professionals to provide better insights in their own words when dealing with periodontal care maintenance patients. 

This study serves as a patient’s behavior management model when treating periodontal maintenance patients and serves to encourage all dental personnel working at FQHCs that unique communication and to spend more time in explaining benefits of periodontal care is needed to guarantee patients compliance within FQHC^17^. 

Active communication is important when treating dental patients within FQHC, as some may not have dental care literacy as described by Akl et al. Patients who have systemic conditions lack the understanding of oral health and what systemic conditions affect oral health. ^3^^,^^7^. This is very important to understand because the load of patients seen in FQHCs are patients with compromised systemic health and poor dental care. ^15^ Thus, dental staff should practice patience and use open ended questions and provide time for the patients to ask questions. It is also important that after disclosing a diagnosis, the dentist should explore the patient's emotional response. Shared decision making empowers patients by inviting them to consider the pros and cons of different treatment options, including no treatment. ^18^

This study also helped to evaluate how dental staff in FQHC feel and how they categorized patients and what can be done to promote a much higher compliance. If dealing with patients who have lack of finances, a sliding fee or discount could be presented. ^12^^,^^18^ In other situations, if there are patients that have language barriers the use of interpreters or the hiring of multilingual staff can also make patients more aware about treatment options and would increase compliance^.(^^19^^,^^20^ For the latter, due to the increased immigrant population been seen at Federal Qualified Health Center in the United States, the multilingual staff could be highly beneficial to better serve our patients. One of the last national surveys’ results presented by Carequest, explained that there is need of empathy and cultural understanding from the provider to improve patients’ interest and compliance in their dental treatments. ^22^

## CONCLUSION

In FQHC, active communication with patients and education in periodontal disease, and knowledge of periodontal health benefits by all dental staff, including front desk receptionists may have a favorable influence in patients periodontal care compliance. It is crucial that all dental staff including the person that schedule their appointments and sets reminders, to have understanding of dental disease, this would help patients to increase with their compliance in periodontal care maintenance.

## References

[1] Impact of the health center program.

[2] Goodman Shepherd J, Lemaster M (2014). Working to improve access to care [Internet] Dimensions of Dental. Hygiene.

[3] Murthy VH (2016). Oral Health in America, 2000 to Present Progress made, but Challenges Remain. Public Health Rep.

[4] (2000). Oral Health in America: A Report of the Surgeon General.

[5] Paquette DW, Bell KP, Phillips C, Offenbacher S, Wilder RS (2015). Dentists&apos; knowledge and opinions of oral-systemic disease relationships relevance to patient care and education. J Dent Educ.

[6] Loe H (1993). Periodontal disease the sixth complication of diabetes mellitus. Diabetes Care.

[7] Akl S, Ranatunga M, Long S, Jennings E, Nimmo A (2021). A systematic review investigating patient knowledge and awareness on the association between oral health and their systemic condition. BMC Public Health.

[8] Hodges KO (2020). Increase patient compliance with SRP. Dimensions of Dental Hygiene.

[9] Kumar A (2023). ISP good clinical practice recommendations for gum care. J Indian Soc Periodontol.

[10] Teutsch C (2003). Patient-doctor communication. Med Clin North Am.

[11] Equalizing access to dental care.

[12] Alvenfors A, Velic M, Marklund B, Kylén S, Lingstrom P, Bernson J (2022). &raquo;,» &reg;,® &sect;,§ &shy;,­ &sup1;,¹ &sup2;,² &sup3;,³ &szlig;,ß &THORN;,Þ &thorn;,þ &times;,× &Uacute;,Ú &uacute;,ú &Ucirc;,Û &ucirc;,û &Ugrave;,Ù &ugrave;,ù &uml;,¨ &Uuml;,Ü &uuml;,ü &Yacute;,Ý &yacute;,ý &yen;,¥ &yuml;,ÿ &para;,¶ Difficult &raquo;,» &reg;,® &sect;,§ &shy;,­ &sup1;,¹ &sup2;,² &sup3;,³ &szlig;,ß &THORN;,Þ &thorn;,þ &times;,× &Uacute;,Ú &uacute;,ú &Ucirc;,Û &ucirc;,û &Ugrave;,Ù &ugrave;,ù &uml;,¨ &Uuml;,Ü &uuml;,ü &Yacute;,Ý &yacute;,ý &yen;,¥ &yuml;,ÿ &para;,¶ dental patients: a grounded theory study of dental staff&apos;s experiences. BDJ Open.

[13] Graetz Christian, Johannes C (2022). Erenthal, Periodontal maintenance compliance individual patient responses and discontinuation. BMC Oral Health.

[14] De Carvalho VF, Okuda OS, Bernardo CC, Pannuti CM, Georgetti MA, De Micheli G, Pustiglioni FE (2010). Compliance improvement in periodontal maintenance. J Appl Oral Sci.

[15] Shue BK, Le H (2009). The framework for patient care at California community health center dental clinics. J Calif Dent Assoc.

[16] Shepherd JG, Locke E, Zhang Q, Maihafer G (2014). Health services use and prescription access among uninsured patients managing chronic diseases. J Community Health.

[17] Rosenbaum S (2011). The Patient Protection and Affordable Care Act implications for public health policy and practice. Public Health Rep.

[18] Hashim MJ (2017). Patient-Centered Communication Basic Skills. Am Fam Physician.

[19] Cobos López I (2019). Traducir para el paciente acercamiento y adaptación como modalidad de traducción. Quaderns de Filologia - Estudis Lingüístics.

[20] Muchacho M (2024). The importance of multilingual communication strategies in dentistry.

[21] Julious SA (2005). Sample size of 12 per group rule of thumb for a pilot study. Pharm Stat.

[22] (2024). What Patients Are Telling Us about Their Oral Health: Insights from the Largest National Survey on Oral Health Equity in America.

